# Extracellular vesicles mediated gastric cancer immune response: tumor cell death or immune escape?

**DOI:** 10.1038/s41419-024-06758-8

**Published:** 2024-05-30

**Authors:** Shuo Yang, Shibo Wei, Fang Wei

**Affiliations:** Department of the Seventh General surgery, The Fourth Affiliated Hospital of China Medical University, Shenyang, 110000̥ Liaoning Province PR China

**Keywords:** Tumour immunology, Cell death, Gastrointestinal cancer

## Abstract

Gastric cancer (GC) is a major global health issue, being the fifth most prevalent cancer and the third highest contributor to cancer-related deaths. Although treatment strategies for GC have diversified, the prognosis for advanced GC remains poor. Hence, there is a critical need to explore new directions for GC treatment to enhance diagnosis, treatment, and patient prognosis. Extracellular vesicles (EVs) have emerged as key players in tumor development and progression. Different sources of EVs carry different molecules, resulting in distinct biological functions. For instance, tumor-derived EVs can promote tumor cell proliferation, alter the tumor microenvironment and immune response, while EVs derived from immune cells carry molecules that regulate immune function and possess tumor-killing capabilities. Numerous studies have demonstrated the crucial role of EVs in the development, immune escape, and immune microenvironment remodeling in GC. In this review, we discuss the role of GC-derived EVs in immune microenvironment remodeling and EVs derived from immune cells in GC development. Furthermore, we provide an overview of the potential uses of EVs in immunotherapy for GC.

## Facts

- GC cells-derived EVs affect infiltrating immune cells in GC;
- Immune cell‐derived EVs can participate in immune regulation of GC cells;
- EVs derived from tumor cells and immune cells have potential clinical applications in immunotherapy of GC.

## Open question

- Which immune cells do GC Cell-derived EVs regulate? Does it promote or inhibit the activity and growth of immune cells? What is the specific mechanism?
- Which immune cells regulate the growth of GC cells through EVs, and what is the specific mechanism?
- How to exploit the immunotherapeutic effects and immunomodulatory mechanisms of EVs in GC to develop drugs?

## Introduction

Gastric cancer (GC) is a major global health issue, being the fifth most prevalent cancer and the third highest contributor to cancer-related deaths [1]. Although current treatment strategies for GC have diversified, there has been limited improvement in the prognosis of patients with advanced GC. Therefore, the discovery of non-invasive biomarkers with high sensitivity and specificity, the exploration of molecular mechanisms driving the development and advancement of GC, and the pursuit of innovative therapeutic approaches are of great importance in improving diagnostic accuracy, treatment efficacy, and patient prognosis for GC [2].

Extracellular vesicles (EVs) are tiny vesicles secreted by living cells into the surrounding microenvironment, playing a crucial role in intercellular communication through paracrine signaling and various other mechanisms [3]. EVs have been implicated in tumor development, cardiovascular diseases, neurodegenerative diseases, and metabolic diseases. They have potential applications as diagnostic markers, therapeutic agents, drug targets, and drug carriers [4–6]. The heterogeneity of extracellular vesicles (EVs) arises from the wide array of cell types and functional states from which they are released, as well as the diverse biological and genetic pathways involved. Consequently, EVs provide a real-time reflection of cellular states, and alterations in exosomal cargoes contribute to distinct biofunctional changes in recipient cells, thereby giving rise to biofunctional heterogeneity [7]. Different sources of EVs have different biological functions due to the different molecules they carry [8, 9]. For example, tumor-derived EVs can induce tumor cell proliferation, change tumor microenvironment and immune microenvironment, and promote tumor invasion and metastasis [10, 11]; EVs derived from immune cells carry molecules that can regulate immune function in the microenvironment and kill tumors [12]. Multiple studies have provided increasing evidence supporting the significant role of EVs in the initiation, progression, and immune evasion of GC [13, 14]. In this review, we explore the involvement of GC-derived EVs in the remodeling of the immune microenvironment. We also examine the impact of EVs derived from immune cells on the development of GC. Furthermore, we provide an overview of the potential applications of EVs in immunotherapy for GC.

## GC cells‐derived EVs communicate with immune cells

**Fig. 1:**
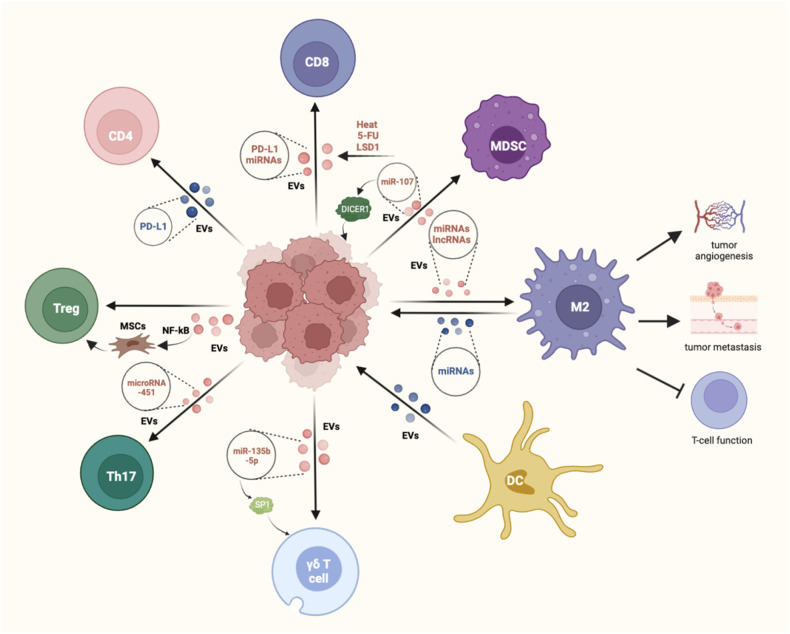
Mechanism and immunomodulatory role of EVs in shuttling between gastric cancer and immune cells. EVs derived from GC cells can act on CD8^+^ T, CD4^+^ T cells, Tregs, γδ T cells, macrophages, NK cells and MDSCs. In addition, EVs derived from macrophages and DCs can affect the immune response of tumors by acting on GC cells.

Emerging studies have revealed a growing body of evidence indicating that GC-derived EVs hold the potential to be valuable indicators indicators of the immunosuppressive status observed in patients with advanced GC, thereby linking to GC prognosis [15, 16]. This chapter aims to explore the impact of EVs derived from GC cells on immune cells that infiltrate the tumor microenvironment (Fig. 1).

### Effects on T cells

#### CD8^+^ T cells

The researchers conducted an investigation into the impact of EVs derived from GC cell lines, specifically MKN-28, MKN-45, and SGC-7901, on immune function. In vitro experiments demonstrated that EVs derived from GC cells had an impact on the secretion of cytokines, including IL-2, IL-6, IL-10, and IFN-γ, in CD8^+^ T cells. Additionally, these EVs hindered the progression of the cell cycle in CD8^+^ T cells, ultimately inducing apoptosis [17]. Moreover, the findings from in vivo studies provide additional evidence that EVs derived from GC decrease the population of CD8^+^ T cells [17]. It is well known that PD-L1 expressed by tumor cells binds to the PD-1 receptor present on T cells to activate the inhibitory signal, thus effectively inhibiting T cell activation [18]. Shin et al. conducted a study involving 99 advanced GC patients. Patients were divided into low exoPD-L1 and high exoPD-L1 groups. Findings showed that the low exoPD-L1 group showed a tendency of longer PFS than the high exoPD-L1 group. Importantly, high exoPD-L1 group showed a significantly lower frequency of CD8+ T cells than the low exoPD-L1 group [19]. This indicates that EV-delivered PD-L1 produced by GC cells can interact with PD-1 on infiltrating T cells, leading to the apoptosis and inactivation of T cells. In addition, basic research has found the same phenomenon. Knockdown of HRS markedly reduced PD-L1 expression in head and neck squamous cells carcinoma (HNSCC) cell-derived sEVs, and these sEVs from HRS knockdown cells showed decreased immunosuppressive effects on CD8+ T cells [20]. HRS, serving as a crucial component of ESCRT-0, plays a pivotal role in the initial recognition and sorting of protein cargo destined for incorporation into ILVs within MVBs [20]. Furthermore, research has unveiled that the phosphorylation of hepatocyte growth factor-regulated tyrosine kinase substrate (HRS) is responsible for driving the secretion of immunosuppressive exosomes bearing PD-L1, thereby limiting the infiltration of CD8+ T cells into tumor tissues [21]. However, the research has yet to elucidate the mechanisms by which exosomes derived from PD-L1-carrying GC cells regulate the infiltration of CD8+ T cells within tumors and the functionality of these infiltrating CD8+ T cells. Conducting exosome tracking experiments can aid in directly observing the uptake of PD-L1+ exosomes by infiltrating CD8+ T cells in GC, thus allowing for an exploration of any functional changes in these CD8+T cells. In addition, the current study has not explored the expression of HRS in gastric cancer tissues, and it is promising to explore whether HRS can mediate the secretion of PD-L1+ EVs by GC to mediate the immune response of CD8+ T cells. Furthermore, the expression of other immune checkpoints, such as TIM3 and LAG and their corresponding ligands, in GC-derived EVs is still unknown. Exploring their role and mechanism in regulating anti-tumor immunity mediated by CD8+ T cells is helpful to search for new therapeutic targets.

Besides the PD-1/PD-L1 axis, miRNAs carried by EVs also contribute significantly to the function of CD8^+^ T cell. Liang et al. found that miR-1290 was highly enriched in GC cells EVs. The researchers observed that miR-1290 facilitated the suppressive effect of GC cells on T cell activation by upregulating PD-L1 through the Grhl2/ZEB1 pathway. Intriguingly, the pivotal role of EV-transmitted miR-1290 as a modulator of tumor immunity was further substantiated in in vivo experiments [22]. In addition, tumor-derived EVs rich in specific miRNAs can induce polarization of T cells towards CD8^+^ T subpopulations in tumor-infiltrating lymphocytes, draining lymph nodes (DLNS), and spleen cells, thereby inducing a beneficial anti-tumor immune response [23]. Similarly, exosome-derived microRNA-433 has been found to inhibit tumorigenesis in non-small cell lung cancer by incremental infiltration of CD8^+^ T cells [24]. This suggests that the different miRNAs carried by tumor-derived EVs may play a dual role in promoting and suppressing cancer in tumors. However, until now, no GC-derived EVs carrying miRNAs have been found to be involved in regulating the infiltration and function of CD8^+^ T cells, so do such EVs-miRNAs related to immune activation exist? It remains to be further explored. The key is to explore the mechanism of miRNAs inducing subgroup polarization of CD8^+^T cells and infiltration of CD8^+^T cells in tumor tissues, which is helpful to further search for mirnas that play an immune activation role in EVs derived from gastric cancer. However, the above studies have not further analyzed the mechanism. Notably, miRNAs were found to regulate the expression of chemokines in tumors, which could help further influence immune cell infiltration [25].

So how to interfere with the secretion and contents of EVs to affect the level and function of tumor-infiltrating CD8^+^ T cells? An early publication revealed that heat-treated ascites from advanced GC patients exhibited elevated levels of heat shock proteins (Hsp70 and Hsp60) compared to untreated ascites-derived EVs in GC patients. Further in vitro investigations indicated that EVs originating from heat-treated malignant ascites possessed the capacity to activate response of tumor-specific cytotoxic T lymphocytes (CTLs) [26]. Later research conducted by Zhang et al. demonstrated that 5-FU treatment promoted the expression of PD-L1 in EVs derived from GC cells, which subsequently contributed to T-cell apoptosis, thereby inducing immunosuppression [27]. A recent study found that histone Lysine-specific demethylase 1 (LSD1) expression in GC samples was negatively correlated with CD8 expression and positively correlated with PD-L1 expression. Subsequent research revealed that the deletion of LSD1 led to a reduction in exosomal PD-L1 and the restoration of T cell function in GC [28]. These findings reveal the role of LSD1 in regulating immune escape in gastric cancer, making LSD1 a potential target for immunotherapy.

#### CD4^+^ T cells

Studies have shown that multiple tumor-derived extracellular vesicles can act on CD4^+^ T cells [29, 30]. As for GC, in a retrospective study, the researchers examined the correlation between exosomal PD-L1 and T cell counts in the plasma of 31 patients with metastatic GC before they underwent chemotherapy [15]. The results showed that exosome PD-L1 levels were negatively correlated with CD4^+^ T cell counts, suggesting a potential association between exosomal PD-L1 and the immunosuppressive status of GC patients [15]. Nevertheless, further investigation is needed to provide direct evidence regarding the impact of GC-derived exosomal PD-L1 on the immune activity of CD4^+^ T cells. In addition, can GC-derived EVs carry other immune checkpoints to affect CD4^+^ T cell immune activation? This will be the future research direction.

Notably, different subpopulations of CD4^+^ T cells give CD4^+^ T cells a dual role in immune regulation of GC. GC cell-derived EVs-treated mesenchymal stem cells (MSCs) enhanced the activation of CD69 and CD25 on the surfaces of T cells [31]. CD25 serves as a surface marker specifically associated with regulatory T cells (Tregs) [32]. Tregs exert a profound immunosuppressive effect, serving to facilitate immune evasion by tumor cells [33]. These findings demonstrate that GC cell-derived EVs stimulate MSCs, leading to the upregulation of tumor-infiltrating Tregs in GC. Additionally, the activation of MSCs by GC-derived EVs is mediated by NF-kB pathway [31]. Aforementioned findings indicate that EVs released by GC cells have the ability in modulating immunosuppression microenvironment via promoting Tregs expansion, thereby facilitating immune evasion by tumor cells. However, it is unclear what content in EVs mediates the amplification of Tregs in GC. Interestingly, a recent study revealed that exosomes from acute myeloid leukemia can transport miR-24-3p to T cells, leading to enhanced Tregs development via NF-kB signaling pathways [34]. In another study, Huang et al. found that exosomal circGSE1 derived from hepatocellular carcinoma (HCC) cells promotes the progression of HCC by inducing Tregs expansion via regulating the miR-324-5p/TGFBR1/Smad3 axis [35]. These findings illustrate the involvement of non-coding RNAs, such as miRNAs and circRNAs, derived from tumor EVs in the regulation of Tregs’ development, thereby impacting the immune response to tumors. Nonetheless, it remains unclear whether EVs derived from GC transport tumor-derived non-coding RNAs responsible for regulating the growth and differentiation of Tregs. This uncertainty underscores the need for targeted studies to identify specific non-coding RNAs within these EVs and understand their functional impacts on Treg behavior.

On the contrary, Liu et al. [36] showed that GC-infiltrating T cells exhibited increased differentiation into T-helper 17 (Th17) cells, which was facilitated by the redistribution of microRNA-451 mediated by EVs. Th17, another subgroup of CD4^+^ T cells, secretes IL-17A, IL-17F, IL-21, IL-22, and CCL20, and expresses Rorc7-encoded master transcription factor RORγt [37], promoting inflammation in response to infection [38, 39]. In contrast to Treg, Th17 has been shown to promote anti-tumor immunity [40]. Combined with the above results, it can be seen that GC-derived EVs can not only lead to immune escape of GC, but also promote anti-tumor immunity in GC. The function of GC-derived EVs is mainly dependent on the contents, so future studies need to continue to discover the different roles of EVs contents in order to search for new targets and biomarkers.

#### γδ T cells

Gamma delta (γδ) T cells, which represent a unique subset of T cells, have a vital role in both innate and adaptive immune surveillance. These cells express a T cell receptor (TCR) consisting of γ and δ chains [41]. Vγ9Vδ2T cells, a prominent subset within γδT cell population, exhibit significant anti-tumor effect [42]. Recent research has demonstrated EVs secreted by GC cells, which carry miR-135b-5p, exert a negative impact on the functionality of Vγ9Vδ2 T cells. These EVs-miR-135b-5p specifically target specificity protein 1 (SP1), leading to impaired functionality of Vγ9Vδ2T cells. Consequently, targeting exosomal miR-135b-5p/SP1 axis presents a promising therapeutic approach to enhance the effectiveness of GC immunotherapy that relies on Vγ9Vδ2 T cells [43]. There are few studies on γδ T cells, which may be the direction of future mining.

### Effects on macrophages

Macrophages are highly prevalent immune-related stromal cells that infiltrate tumors. They can be categorized into two main phenotypes. One of the two phenotypes is M1 macrophages, also known as classically activated macrophages, which predominantly display anti-inflammatory and antitumor properties [44]. On the other hand, alternatively activated macrophages, known as M2 macrophages, possess immunosuppressive properties and the ability to promote tumor growth [45]. The transformation of macrophages into the M2 phenotype plays a crucial role in shaping the suppressive immune microenvironment, thereby facilitating tumor immune evasion [46, 47]. Increasing evidence indicates that tumor-derived EVs play a significant role in promoting the M2 polarization of tumor-associated macrophages (TAMs), thereby contributing to the formation of an immunosuppressive microenvironment within the tumor [48]. In a similar manner, EVs isolated from GC cells, as well as malignant ascitic fluid, promote the polarization of macrophages derived from peripheral blood mononuclear cells into an M2-like phenotype. This was evidenced by the altered morphology of the macrophages and the increased expression of CD163/206, which are markers associated with the M2-like phenotype [49]. Numerous studies have consistently shown that GC-derived EVs promote tumor progression by inducing polarization of M2-like macrophages. Notably, one such study highlighted the specific involvement of miR-151-3p carried by GC EVs in inducing the phenotypic polarization of macrophages, thereby facilitating tumor progession [50]. Additionally, tumor-derived EVs-lncRNAs, such as ElNF1-AS1 [51], HCG18 [52], and MIR4435-2HG [53], also affect the progression of GC by promoting M2 polarization of macrophages. In addition to non-coding RNAs, metabolic adaptations are also associated with the polarization of macrophages [54]. A previous investigation revealed that EVs derived from HCC, carrying Pyruvate kinase M2 isoform (PKM2), foster the progression of HCC by driving M2 polarization of macrophages and reconfiguring the tumor immune microenvironment [55]. It is important to highlight that a recent study identified a similar phenomenon in GC. Wu et al. discovered that gastric cancer cells can deliver exosomal PKM2 to macrophages, leading to the differentiation of macrophages into the M2 subtype, consequently promoting the progression of GC [56]. Overall, tumor cell-derived exosome PKM2 promotes macrophage differentiation through glycolytic reprogramming. PKM2 is just one of the glycolytic enzymes. Future research can delve into whether GC transfers other glycolytic enzymes to macrophages through EVs, thereby reshaping macrophage glycolytic metabolism and influencing macrophage differentiation. In a study involving patients with neuroblastoma, glycolytic enzymes PKM2 and Hexokinase II (HK2) were detected in circulating EVs isolated from blood using mass spectrometry [57]. Therefore, future research could utilize mass spectrometry to explore the differential expression of metabolic enzymes in EVs secreted by GC cells, potentially providing new insights into the metabolic alterations associated with cancer progression.

In addition, the activation of the NF-kB signaling pathway has been identified as a key mediator in the induction of M2 macrophages by tumor-derived EVs, contributing to the progression of GC. This finding offers a promising therapeutic avenue for GC by targeting the interaction between EVs and macrophages within the tumor microenvironment, thereby disrupting the pro-tumorigenic effects of EV-mediated M2 polarization [58]. The study underscores the significance of the NF-kB signaling pathway in mediating M2 macrophage induction by tumor-derived EVs and its potential as a therapeutic target for GC. However, the study falls short in revealing the specific components in exosomes that are involved in activating the NF-kB signaling pathway. In recent research, High Mobility Group Box 1 (HMGB1) was found to be highly expressed in EVs derived from GC. It interacts with the transcription factor POU2F1 in macrophages, subsequently inhibiting the transcriptional activity of p50, leading to the inactivation of the NF-κB signaling pathway. This process induces polarization towards M2-like macrophages. The study also confirmed in vivo that exosomal HMGB1 inhibits the growth of GC, suggesting a complex role where HMGB1 might modulate tumor microenvironment dynamics to influence cancer progression [59]. Moreover, a large number of studies have suggested that JAK/STAT is a key node in macrophage polarization [60]. However, further investigations are essential to ascertain whether it mediates the regulation of macrophage polarization by GC-derived exosomes. Notably, exosomal miR-200b-3p from HCC has been found to induce M2-type polarization in macrophages by JAK/STAT signaling pathway, facilitating tumor progression [61]. More importantly, miR-200b-3p has also been identified as highly expressed in GC obstructions and possesses oncogenic properties [62]. Additionally, Galectin-1 has been proven to influence macrophages through exosomes, activating the JAK/STAT signaling pathway and inducing M2 polarization [63]. And Galectin-1, highly expressed in GC, promoted tumor progression [64]. The findings further affirm that GC may utilize EVs to activate the JAK/STAT signaling pathway, thus promoting M2 polarization in macrophages. Future research should focus on examining whether GC-derived oncogenic factors can influence the JAK/STAT pathway in macrophages through EVs, inducing M2 polarization. The experimental key lies in detecting the expression of relevant molecules in EVs and using tracer techniques to observe if they are carried by EVs into macrophages. Additionally, integrating multi-omics techniques such as transcriptomics and proteomics could help identify more GC-derived factors capable of delivering and activating the JAK/STAT pathway via extracellular vesicles.

M2 macrophages promote tumor progression by inducing tumor angiogenesis [65], metastasis [66], and regulating T-cell function [67]. Therefore, numerous studies have thus investigated the mechanisms by which EVs induce phenotypic changes in macrophages, contributing to tumor progression. Qiu et al’s study demonstrated that GC cells released EVs-miR-519a-3p enhanced liver metastasis through the activation of intrahepatic M2-like macrophages and subsequent induction of angiogenesis. The research findings emphasized the significance of exosomal miR-519a-3p in the activation of the MAPK/ERK pathway through its targeting of DUSP2. This activation subsequently leads to M2-type polarization of TAM and contributes to the promotion of angiogenesis [68]. Additionally, M2-like macrophage-mediated angiogenesis accelerates liver metastasis of GC and promotes intrahepatic premetastatic niche formation [68]. In addition to liver metastasis, GC-derived EVs also facilitates pulmonary metastasis by activating ERK-mediated M2-like macrophages polarization [69]. As for regulating T-cell function, Wang et al. demonstrated that GC-derived EVs play a crucial role in modulating CD8^+^ T-cell function through the education of monocytes. Specifically, these EVs were found to induce the differentiation of monocytes into PD1^+^ TAMs, exhibiting both M2 phenotypic and functional characteristics. The presence of these TAMs resulted in the suppression of CD8^+^ T-cell function, establishing an immunosuppressive microenvironment that facilitates the progression of GC [70]. Given that GC-derived EVs can induce PD1+ TAMs, it raises the question of whether targeting PD1 inhibition can suppress TAM-mediated cancer immune evasion. Future studies could investigate the therapeutic effectiveness of combining PD1 monoclonal antibodies with exosome inhibitors in animal models, using flow cytometry to analyze the infiltration of PD1+ TAMs. Additionally, examining the infiltration of PD1+ TAMs in the GC tissues of patients treated with PD1 monoclonal antibodies could help validate this hypothesis.

While the majority of studies have demonstrated the ability of GC-derived EVs to induce M2-type polarization of tumor-infiltrating macrophages, it is important to acknowledge that EVs can also exert inhibitory effects on such polarization. For example, Song et al. [71]. discovered that exosomal hsa_circ_0017252 plays a crucial role in suppressing the development of gastric cancer by inhibiting the polarization of macrophages towards the M2 phenotype. Although there are few studies on the inhibitory effect of EVs in macrophage M2 type polarization, these findings open new horizons in the search for inhibitory EVs.

Get together, present findings support the dual role of GC-derived EVs in shaping the macrophage-based immune microenvironment, and the challenge ahead is to discover the balancing mechanism of the dual roles.

### Effects on NK cells

Natural Killer (NK) cells, as integral components of the immune system, play a crucial role in surveilling and eliminating tumor cells. Emerging evidence suggests that EVs derived from gastric cancer GC cells actively participate in intercellular communication with NK cells. The study reveals that, after prolonged exposure to exosomes released by gastric cancer cells, mice developed an immunosuppressive tumor microenvironment in the lung, characterized by a significant reduction in NK cell levels [17], creating an immunosuppressive condition for metastatic niche formation in the lung. While the study sheds light on the involvement of NK cells in the communication with GC cells via EVs, there is a notable gap in our understanding regarding the underlying mechanisms responsible for the reduction in NK cell levels induced by EVs from GC cells. Additionally, the specific contents within these EVs that contribute to shaping the NK cell-mediated immunosuppressive microenvironment remain elusive. To address these knowledge gaps, future research endeavors could focus on elucidating the intricate molecular mechanisms mediating the impact of GC-derived EVs on NK cells. Investigating the cargo of these EVs, such as specific proteins, miRNAs, or other bioactive molecules, may unveil key regulators of NK cell function and shed light on potential therapeutic targets. Moreover, exploring the dynamic changes in the tumor microenvironment over time and deciphering the reciprocal communication between EVs and NK cells could provide a more comprehensive understanding of the immunomodulatory processes involved. Ultimately, unraveling these aspects holds great promise for developing targeted interventions aimed at modulating the interplay between GC cells, EVs, and NK cells to enhance anti-tumor immune responses and inhibit metastatic progression.

### Effects on myeloid-derived suppressor cells (MDSCs)

MDSCs are a key component of the tumor microenvironment and exert immunosuppressive effects, promoting tumor progression and potentially impeding the efficacy of immunotherapy interventions [72]. Earlier research has demonstrated that tumor-derived EVs are internalized by MDSCs, and the cargo they carry has the ability to enhance the accumulation and activation of MDSCs, thereby facilitating tumor advancement [73, 74]. In a groundbreaking study, it was recently shown that GC-secreted EVs play a crucial role in delivering miR-107 to host MDSCs. This delivery mechanism leads to the expansion and activation of MDSCs by targeting DICER1 and PTEN genes. These findings present an exciting opportunity for the development of novel therapeutic targets for GC [75]. Currently, there is limited research available regarding the impact of GC-derived EVs on MDSCs. The study conducted by Ren et al. offers valuable insights and lays the foundation for future investigations in this area.

In future research endeavors, there should be a focus on delving into more molecular-level mechanisms to ascertain the impact of GC-derived EVs on MDSCs, facilitating a deeper understanding of specific regulatory networks. Interestingly, EVs derived from glioblastoma that contain basic leucine zipper ATF-like transcription factor 2 (BATF2) have been discovered to inhibit the chemotaxis and recruitment of MDSCs, thus preventing the formation of an immunosuppressive microenvironment typically sculpted by MDSCs [76]. This finding suggests that although tumor-derived EVs predominantly promote tumor immune escape by activating and expanding MDSCs, they can also exert inhibitory effects on MDSCs, depending largely on the specific contents of the EVs [77]. This opens up a new direction for future research, specifically to explore whether EVs derived from GC exert dual effects on MDSCs, potentially both promoting and inhibiting their functions depending on the molecular cargo they carry.

## Immune cell‐derived EVs communicate with GC cells

A growing body of research has highlighted the involvement of immune cell-derived EVs in tumorigenesis. These studies have explored EVs originating from various immune cell types, such as T cells [78], macrophages [79], NK cells [80] and dendritic cells (DCs) [81]. The present study reported that macrophage and DC-derived EVs are involved in the immune regulation of GC (Fig. 1).

### Macrophage-derived EVs

Fluorescence microscopy was utilized to observe the uptake of EVs derived from M2 macrophages by GC cells in co-culture experiments. The results revealed that EVs derived from TAMs play a role in promoting the progression of GC. This effect was attributed to their regulation of the MAPK signaling pathway and the upregulation of PD-L1 expression in GC cells [82]. Importantly, contrasting results were observed in a study focusing on EVs derived from M1 macrophages. The pivotal findings of this study indicate that M1 macrophage-derived EVs, specifically carrying miR-16-5p, possess an inhibitory impact on the progression of GC. This effect is attributed to their ability to activate the T cell immune response by downregulating PD-L1 expression in GC cells [83]. Collectively, these two studies provide compelling evidence that macrophages possess the ability to modulate tumor growth through the targeting of PD-L1. Furthermore, Yang et al. have unveiled additional mechanisms through which EVs derived from M2 macrophages promote tumor progression, as their in vivo and in vitro findings compellingly demonstrate the EVs’ capability to induce the proliferation and tumorigenesis of GC cells [84]. Additionally, miR-487a, which is abundantly present in M2 macrophage-derived EVs, was identified to play a crucial role in their functions. It was discovered that miR-487a exerts its effects by targeting T-cell intracellular antigen-1 (TIA1), a protein widely implicated in cancer progression [85]. These new findings are significantly important for the development of diagnostic and therapeutic approaches for gastric cancer. The potential of GC-derived EVs to contribute to innovative methods for GC diagnosis and treatment could lead to improved outcomes for patients. This underscores the importance of further exploring the role and mechanisms of these EVs in influencing the tumor microenvironment and their direct impacts on cancer progression and response to therapies.

In line with the preceding section, EVs derived from macrophages contribute to the metastasis of GC. Specifically, EVs derived from M2 macrophages facilitate the migration of GC cells by transferring functional Apolipoprotein E in in vitro studies. The underlying mechanism involves the mediation of the PI3K-AKT signaling pathway, which is responsible for the pro-migratory effect of macrophage-derived exosomal ApoE on GC cells [46]. A subsequent investigation delved deeper into the characterization of EV contents. Functional analyses uncovered that EVs carrying miR-223 derived from M2 macrophages facilitated the metastasis of GC cells through the involvement of the PTEN-PI3K/AKT pathway. This study sheds light on the active components of EVs and their role in promoting GC metastasis [86]. The above-mentioned research has unveiled the crucial roles of signaling pathways such as PI3K-AKT and PTEN-PI3K/AKT in the process, making the design of targeted drugs aiming to block or intervene in the migration of GC cells through these pathways a potential therapeutic strategy.

Interestingly, EVs derived from M2-polarized macrophages have shown the ability to induce resistance in GC cells to multiple drugs, including cisplatin (DDP), doxorubicin, and oxaliplatin (OXA). Intriguingly, a previous investigation uncovered that the transfer of miR-21 from tumor-associated macrophages via exosomes confers DDP resistance in GC cells. These findings highlight the role of M2 macrophage-derived EVs in mediating drug resistance in GC [87]. Another study revealed additional evidence indicating the involvement of exosomal miR-588 derived from M2 macrophages in contributing to the resistance of GC cells against cisplatin (DDP). The study demonstrated that miR-588 had the ability to target cylindromatosis (CYLD) specifically within GC cells. Notably, when CYLD was depleted, it resulted in the reversal of the regulatory effects of miR-588 inhibition on cell proliferation and apoptosis in gastric cancer cells exposed to DDP [88]. Furthermore, it was discovered that M2 macrophage-derived EVs containing miR-223 played a role in promoting doxorubicin resistance in GC cells. This resistance mechanism was attributed to the inhibition of F-box and WD repeat domain-containing 7 (FBXW7), a crucial component of the SCF-type ubiquitin ligase complex responsible for substrate recognition [89]. Oxaliplatin (OXA), a third-generation platinum-based anticancer drug with similarities to cisplatin, has been implicated in resistance mechanisms. Specifically, a circular RNA called circ-0008253, found within M2 macrophage-derived EVs, has been observed to undergo direct transfer from M2 macrophages to gastric carcinoma cells [90]. This study holds significant implications for a deeper understanding of drug resistance in GC. By highlighting the multifaceted role of M2-polarized macrophage-derived EVs in GC drug resistance, involving different drugs and mediating mechanisms with multiple non-coding RNAs, it offers a novel perspective on GC treatment. A promising future research direction is to explore whether, in addition to non-coding RNAs, proteins can be transported by EVs and participate in the regulation of chemotherapy resistance in GC. A study in ovarian cancer might offer valuable insights for us, as it revealed that EVs derived from TAMs encapsulate GATA-binding protein-3 (GATA3), which promotes chemotherapy resistance in ovarian cancer cells to DDP by upregulating the CD24/Siglec-10 axis [91]. This provides a theoretical feasibility for further investigating how macrophages induce chemotherapy resistance in GC through the secretion of proteins, in addition to non-coding RNAs. This line of research could reveal new mechanisms by which macrophages contribute to the chemoresistance of GC, potentially leading to novel therapeutic strategies to counteract this resistance.

### DC-derived EVs

DCs are highly efficient professional antigen-presenting cells (APCs) in the body, known for their exceptional ability to uptake, process, and present antigens. Immature DCs display a remarkable migratory capacity, while mature DCs play a pivotal role in activating naive T cells. DCs serve as a central and indispensable component in initiating, regulating, and sustaining the immune response [92]. DCs possess the capability to capture antigens generated and released during tumorigenesis, subsequently presenting tumor antigens to naive CD8^+^ T and CD4^+^ T cells through major histocompatibility complex (MHC)-I and MHC-II molecules. This antigen presentation process serves as a trigger to initiate and activate an effective anti-tumor immune response [93, 94].

DC-derived EVs, small lipid vesicles, have demonstrated their potential in stimulating antitumor immune responses in both mouse models and clinical trials [95]. In a previous study, it was discovered that EVs derived from DCs have the capacity to enhance the proliferation of T lymphocytes in vitro, leading to the generation of potent antitumor immune responses in vivo [96].

Mature DC-derived EVs exhibit elevated expression of tumor antigen peptide-MHC complexes and crucial costimulatory factors like CD86 on their surface. These EVs effectively present these complexes to immune cells, stimulating the activation of TAA-specific effector T cells [97, 98]. In the context of GC, Wang et al. found that hyperthermic CO_2_ (HT-CO_2_) treatment of DC-derived EVs exhibited notable inhibitory effects on the proliferation of GC cells and induced apoptosis in a time-dependent manner. Furthermore, HT-CO_2_ treatment of DC-derived EVs led to a reduction in the expression of HSP70 and demonstrated suppressed tumor growth in nude mice models [99]. HSP70, a prominent component of EVs, plays a vital role in cellular processes. Earlier research has presented evidence supporting the notion that both hematopoietic and tumor cells possess the capability to release HSPs into the bloodstream through diverse mechanisms, including exosome-mediated secretion, granule-mediated secretion, and lipid raft-mediated exocytosis [100]. Extracellular HSPs have been found to activate innate immune responses by binding to Toll-like receptors (TLRs). This interaction triggers a cascade of immune signaling events, leading to the activation of innate immune cells and the initiation of immune responses [101]. The above-mentioned study provides crucial insights into the role of DC-derived EVs in anti-tumor immunity. However, the article discusses the suppressive effects of DC-derived EVs on GC but does not detail the specific components within these EVs. It is noteworthy that DC-derived EVs, following infection by Toxoplasma gondii, can promote macrophage M1 polarization through the carriage of miR-155-5p, thereby inhibiting the progression of colon cancer [102]. Another study identified a significant overexpression of the heat shock protein HSC73 in exosomes derived from dendritic cells, enhancing the immunogenicity of DCs [103]. These findings indicate that DC-derived exosomes can transport both RNA and proteins to participate in activating tumor immune responses. An in-depth analysis of EV components, including proteins, RNA, and other constituents, should be conducted to identify key bioactive molecules. This approach will contribute to a more comprehensive understanding of the mechanisms underlying the inhibitory effects observed in GC cells.

## Potential application of EVs in GC immunotherapy

**Fig. 2:**
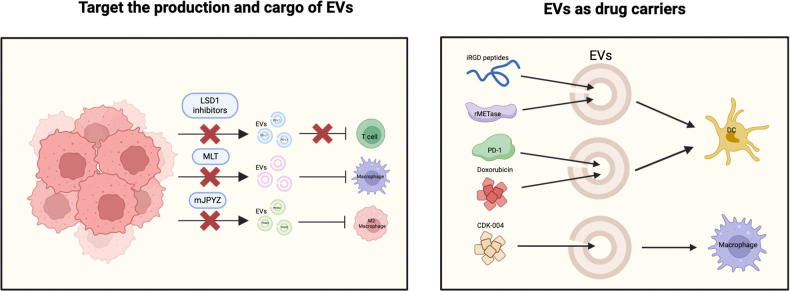
Potential application of EVs in GC immunotherapy. The potential applications of EVs in GC immunotherapy, encompassing the targeting of EVs’ production and cargo, as well as exploring their role as effective drug carriers.

This section will elaborate on the potential applications of EVs in GC immunotherapy, encompassing the targeting of EVs’ production and cargo, as well as exploring their role as effective drug carriers (Table 1 and Fig. 2).

**Table 1:** Potential application of EVs in GC immunotherapy.

Potential drug or nano-preparation	Target immune cell	Treatment strategy based on EVs	Ref.
LSD1 inhibitors	T cell	Target the production and cargo of EVs	[28]
MLT	Macrophage	Target the production and cargo of EVs	[14]
mJPYZ	Macrophage	Target the production and cargo of EVs	[56]
iRGD-Exos-rMETase	DCs	EVs as drug carriers	[107]
EVs-PD-1 antibodies and doxorubicin	DCs	EVs as drug carriers	[108]
CDK-004	Macrophage	EVs as drug carriers	NCT05375604

### Target the production and cargo of EVs

The above chapter illustrates that GC and immune cell-derived EVs can reshape the tumor immune microenvironment, so intervening the production and contents of EVs can be a strategy for GC immunotherapy. Since EVs play a dual role in the progression of GC, it is necessary to search for precise targeted regulatory strategies. A recent study demonstrated that the deletion of LSD1 leads to a reduction in exosomal PD-L1 levels and restores T-cell response in GC [28], which suggests that targeting LSD1 could be a promising strategy to inhibit the growth of GC. Currently, there are nine LSD1 inhibitors, including tranylcypromine, ORY-1001, ORY-2001, GSK-2879552, IMG-7289, INCB059872, TAK-418, CC-90011, and SP-2577, that have entered clinical trials for this finding suggests that targeting LSD1 could be a promising strategy to inhibit the growth of GC. Currently, there are nine LSD1 inhibitors, including tranylcypromine, ORY-1001, ORY-2001, GSK-2879552, IMG-7289, INCB059872, TAK-418, CC-90011, and SP-2577, that have entered clinical trials for disease treatment [104]. Future research can focus on investigating the inhibitory effects of currently available LSD1 inhibitors on GC growth, as well as assessing their safety profiles. Moreover, the discovery has been made that melatonin enhances the generation of EVs, which in turn promote the recruitment of CD8^+^ T cells to the tumor site, ultimately leading to the inhibition of GC growth [14]. Melatonin (MLT), a hormone known for its potential anti-tumor properties, holds promise as a therapeutic agent for GC. Interestingly, the intriguing properties of MLT extend to its ability to regulate the expression of PD-L1 in macrophages through the modulation of exosome-associated proteins and microRNAs [14]. Therefore, targeted regulation of EVs production and selective loading of its contents may be a novel strategy for GC immunotherapy. Significantly, Traditional Chinese Medicine (TCM) has demonstrated its ability to modulate the cargo of EVs. One notable example is the Modified Jianpi Yangzheng decoction (mJPYZ), an empirical formulation in TCM that has exhibited remarkable efficacy in extending the survival of patients with advanced GC [105]. Wu et al. found that mJPYZ can reduce the content of exosomal PKM2 in GC cells, thereby reducing the transport of exosomal PKM2 from tumor cells to macrophages, and alleviating the differentiation of M2-TAM induced by exosomal PKM2 in the tumor microenvironment, ultimately inhibiting the progression of GC [56]. Findings introduce a novel opportunity for the advancement of drugs targeting EVs. It presents a fresh perspective for the development of EVs-targeting drugs, offering a broader range of therapeutic options. In addition to Western medicines, the well-established anti-gastric cancer effects of mature TCM highlight their potential as EVs-targeting immunotherapy drugs for GC.

### EVs as drug carriers

Multiple types of EVs have demonstrated their efficacy as drug carriers, possessing both functional therapeutic properties and targeted delivery capabilities [106]. In GCs, EVs can also be used as targeted drug delivery vehicles. An illustrative example involves the fusion of immature dendritic cell-derived EVs (Exos), with internalizing RGD (iRGD) peptides, resulting in the formation of a specialized type of tumor-targeting Exos called iRGD-Exos. Furthermore, to enhance therapeutic efficacy, recombinant methioninase (rMETase) was efficiently loaded into the iRGD-Exos via electroporation, giving rise to iRGD-Exos-rMETase. This innovative approach represents a compelling anti-GC therapy that capitalizes on the tumor-targeting capabilities of iRGD-Exos to deliver rMETase specifically to tumor tissue, thereby offering an effective treatment strategy for GC [107]. In another study, the vector formed by the fusion of DCs-derived EVs and induced pluripotent stem cells derived EVs that could load PD-1 antibodies and doxorubicin, effectively inhibiting GC expansion process through synergistic chemotherapy and immunotherapy, and showed the ability to improve prognosis [108]. Importantly, a Phase I clinical trial of EVs-supported nanomedical targeting GC-infiltrated macrophages has been conducted (NCT05375604). In this study, scientists developed a promising therapeutic candidate called CDK-004, which involves the use of cell-derived EVs loaded with a synthetic lipid-tagged oligonucleotide. CDK-004 has been designed to facilitate the targeted delivery of an antisense oligonucleotide (ASO) against STAT6 to myeloid cells. The aim is to induce a repolarization of macrophages from an immune-suppressive M2 phenotype to a proinflammatory M1 phenotype. This innovative approach holds significant potential for achieving substantial antitumor activity as a standalone treatment, which has not been observed with other pathway inhibitors currently available. The study has now been terminated, but the results have not yet been published, and it is believed that the results of the study can be seen in the near future to guide the further work of researchers.

## Conclusions and future challenges

EVs, as a rapidly growing field, have demonstrated an immense impact on the initiation, progression, prognosis, and treatment of GC. GC ranks as the fifth most prevalent cancer and the third leading cause of cancer-related deaths worldwide. Thus, it is imperative to further investigate the potential of immunotherapy in GC and uncover the underlying mechanisms. Given the significant involvement of EVs in cancer immunotherapy, ongoing research is delving into the role of GC-derived EVs in reshaping the immune microenvironment and immune cell-derived EVs in the development of GC. This comprehensive exploration aims to highlight the potential application of EVs in the field of GC immunotherapy.

This study provides a comprehensive analysis of the extensive research conducted on the regulatory effects of tumor-derived EVs on immune cell differentiation, maturation, proliferation, and function. Numerous studies have demonstrated the impact of tumor-derived EVs on various immune cell populations, including CD8^+^ T cells, CD4^+^ T cells, γδ T cells, macrophages, and MDSCs. These findings shed light on the intricate mechanisms by which tumor-derived EVs modulate GC progression. Additionally, the involvement of macrophage and DC-derived EVs in the immune regulation of GC has also been documented. Moreover, leveraging their inherent capacity for long-distance communication and exceptional biocompatibility, EVs hold promising potential for application in GC immunotherapy. In the current study, therapeutic approaches focusing on the modulation of EVs production and contents, as well as utilizing EVs as delivery vehicles, have been proposed. However, several challenges still exist, impeding the broader application of EVs in GC immunotherapy. Improving the accuracy and standardization of EVs extraction methods is a primary concern. Additionally, there is an urgent need to enhance the efficiency of loading drugs or antigens into EVs and develop more convenient methods for evaluating the loading efficiency. Pan et al. developed passion fruit-like Exo-PMA/Au-BSA@Ce6 nanocarriers using a real-time electroporation approach to effectively load multifunctional PMA/Au-BSA@Ce6 nanoparticles with EVs obtained from fresh urine samples of numerous GC patients. In this system, the engineered Exo-PMA/Au-BSA@Ce6 nanovehicles demonstrated efficient internalization into cancer cells. Additionally, the nanovehicles exhibited a unique membrane structure and antigens that could delay endocytosis by macrophages and prolong their circulation time in the bloodstream [109]. The combination of multifunctional nanoparticles and EVs provides a new strategy to overcome the low loading efficiency of EVs. It is believed that there will be more ways to overcome these difficulties in the future, thus promoting the development of EV-dependent drugs or EV-targeting drugs in the future, and making it one of the potential strategies to treat GC.
